# Unravelling the mechanisms of underweight in Parkinson’s disease by investigating into the role of gut microbiome

**DOI:** 10.1038/s41531-023-00587-w

**Published:** 2024-01-24

**Authors:** Ling-Chieh Shih, Ru-Jen Lin, Yan-Lin Chen, Shih-Chen Fu

**Affiliations:** 1 Taipei Veterans General Hospital, Taipei, Taiwan, ROC; 2 National Taiwan University Hospital Hsin-Chu Branch, Hsin-Chu, Taiwan, ROC; 3 Institute of Statistics, National Yang Ming Chiao Tung University, Hsin-Chu, Taiwan, ROC; 4 Department of Life Science, National Dong Hwa University, Hualien, Taiwan, ROC

**Keywords:** Parkinson's disease, Microbiology

## Abstract

Approximately half of patients with Parkinson’s disease (PD) suffer from unintentional weight loss and are underweight, complicating the clinical course of PD patients. Gut microbiota alteration has been proven to be associated with PD, and recent studies have shown that gut microbiota could lead to muscle wasting, implying a possible role of gut microbiota in underweight PD. In this study, we aimed to (1) investigate the mechanism underlying underweight in PD patients with respect to gut microbiota and (2) estimate the extent to which gut microbiota may mediate PD-related underweight through mediation analysis. The data were adapted from Hill‐Burns et al., in which 330 participants (199 PD, 131 controls) were enrolled in the study. Fecal samples were collected from participants for microbiome analysis. 16S rRNA gene sequence data were processed using DADA2. Mediation analysis was performed to quantify the effect of intestinal microbial alteration on the causal effect of PD on underweight and to identify the key bacteria that significantly mediated PD-related underweight. The results showed that the PD group had significantly more underweight patients (body mass index (BMI) < 18.5) after controlling for age and sex. Ten genera and four species were significantly different in relative abundance between the underweight and non-underweight individuals in the PD group. Mediation analysis showed that 42.29% and 37.91% of the effect of PD on underweight was mediated through intestinal microbial alterations at the genus and species levels, respectively. Five genera (Agathobacter, Eisenbergiella, Fusicatenibacter, Roseburia, Ruminococcaceae_UCG_013) showed significant mediation effects. In conclusion, we found that up to 42.29% of underweight PD cases are mediated by gut microbiota, with increased pro-inflammatory bacteria and decreased SCFA-producing bacteria, which indicates that the pro-inflammatory state, disturbance of metabolism, and interference of appetite regulation may be involved in the mechanism of underweight PD.

## Introduction

Parkinson’s disease (PD) is a neurodegenerative disease with a worldwide prevalence of 0.3% in the population ages 40 and older^1^. Symptoms of PD include motor (tremor, bradykinesia, rigidity, and postural instability) and non-motor (constipation, rapid eye movement sleep behavior disorder, depression, and weight loss) symptoms^2–4^. Approximately 52–65% of patients with PD suffer from unintentional weight loss^5^, with a mean loss of 3–6 kg during the disease process^6^. PD patients with weight loss symptoms tend to have a lower body mass index (BMI) and are underweight^7^. Weight loss and underweight could complicate the clinical course of PD, leading to poor prognosis^8–10^ and a poor quality of life^11^. For instance, clinically significant weight loss (loss >5% from baseline) occurring within one year of PD diagnosis was found to be associated with an increased risk of dependency (requiring help with basic activities of daily living), dementia and death^10^. Weight loss has also been found to be a risk factor for osteoporosis and fractures in PD patients^8^. A 6 months follow-up study showed that PD patients with a decreased BMI had lower cognitive function and faster rates of cognitive decline than patients with a stable BMI^9^. The economic burdens of PD in the United States was estimated as $51.9 billion and the aforementioned complications in PD patients might contribute to both medical and non-medical costs (including caregiver burdens, home modification, disability income provided to PD patients)^12^. However, to date, there are no appropriate interventions or prevention methods specifically tailored for weight loss in PD^13^. Therefore, understanding the mechanism of underweight in PD and identifying potential treatment targets are crucial.

The gut microbiota is a complex ecological community composed of 100 trillion of microbes, which influences both normal physiology and disease susceptibility through its metabolic activities and host interactions^14^. Alteration of the gut microbiota can disturb gut barrier function, leading to local and systemic inflammation in the human body, and this mechanism has been shown to be involved in the development of irritable bowel syndrome^15^, inflammatory bowel disease^16^ and autoimmune disease^17^. In addition, the gut microbiota can affect insulin signaling and energy metabolism, which has been proposed to be involved in the development of metabolic syndrome, obesity, and type 2 diabetes^18^. Recent studies have found that probiotic supplementation can increase muscle mass and the proportion of type I muscle fibers in mice^19^. Probiotic supplementation could also improve handgrip strength and exercise performance in elderly and cancer patients^20,21^. Growing body of evidence has resulted in the proposal of a ‘gut–muscle axis’^22–24^, in which changes in muscle mass or skeletal muscle metabolism induced by gut microbiota can consequently affect body weight. Several neurological and psychiatric disorders, such as depression, schizophrenia, Alzheimer’s disease, and PD, are correlated with gut microbiota alteration^25^. Many studies have shown that PD is associated with dysbiosis of the intestinal microbiota, suggesting that gut microbiota may play a role in PD-related underweight.

To date, only one pilot study has investigated the role of gut microbiota in PD-related underweight, in which dissimilarity of gut microbiota profiles was found between PD patients with and without weight loss^26^. However, the mechanism by which gut microbiota contribute to underweight in PD, as well as the extent of its impact on PD-related weight loss, remains unknown. In this study, we hypothesized that the impact of PD on underweight individuals may be mediated by gut microbiota. Therefore, our aims were to (1) investigate the mechanism underlying underweight in PD patients with respect to gut microbiota and (2) estimate the extent to which gut microbiota may mediate PD-related underweight through mediation analysis.

## Results

### Demographic variables comparison

We compared the general characteristics between the PD (*N* = 199) and control (*N* = 131) groups. The results are summarized in Table 1. The PD group was significantly younger (68.4 in PD vs 70.4 control group; *p* = 0.033) and had more male participants (66.8% vs. 39.7% in control group; *p* < 0.001). After controlling for age and sex, there were no significant differences between the PD and control groups in the categories of race, geographic location, alcohol consumption amount, coffee consumption amount, smoking, diet habits (eating fruits or vegetables daily, eating grains daily, eating meat daily), presence of other neurological diseases, and presence of cancer. After controlling for age and sex, the PD group had a significantly lower BMI (26.4 in PD vs 28.3 in control group; adjusted *p*-value < 0.001) and a higher percentage of underweight individuals (4.5% in PD vs. 1.5% in the control group; adjusted *p*-value = 0.011). To understand whether underweight in the PD group was associated with mental health, gastrointestinal disease, gastrointestinal discomfort, and dietary habits, we compared several characteristics between underweight (*N* = 9) and non-underweight (*N* = 183) individuals in the PD group. The results are summarized in Table 2. There were more female participants (77.8% underweight vs. 30.6% non-underweight; *p* = 0.01) in the underweight PD group. There were no significant differences in age, depression, anxiety, inflammatory bowel disease, irritable bowel disease, constipation, gastrointestinal symptoms other than constipation, dietary habits (eating fruits or vegetables daily, eating grains daily, eating meals daily, eating yogurt daily), and probiotics intake between underweight and non-underweight individuals in the PD group.

**Table 1:** Comparing patients with and without Parkinson’s disease (PD) on demographic variables.

	Controls (*N* = 131)	PD (*N* = 199)	*P*-value (adjusted *P*-value)
Age			0.033
Mean (SD)	70.4 (8.60)	68.4 (9.14)	
Sex			<0.001
Female	79 (60.3%)	66 (33.2%)	
Male	52 (39.7%)	133 (66.8%)	
Race			0.264 (0.139)
White	131 (100%)	195 (98.0%)	
Black or African American	0 (0%)	1 (0.5%)	
More Than One Race	0 (0%)	3 (1.5%)	
Location			0.147 (0.310)
Seattle, WA	57 (43.5%)	92 (46.2%)	
Albany, NY	61 (46.6%)	75 (37.7%)	
Atlanta, GA	13 (9.9%)	32 (16.1%)	
Body mass index (BMI)			0.009 (<0.001)
Mean (SD)	28.3 (5.69)	26.4 (5.32)	
Missing	4 (3.1%)	7 (3.5%)	
Alcohol amount			0.017 (0.081)
2 or more drinks a week	39 (29.8%)	64 (32.2%)	
Less than 2 drinks a week	53 (40.5%)	51 (25.6%)	
None	38 (29.0%)	79 (39.7%)	
Missing	1 (0.8%)	5 (2.5%)	
Coffee amount			0.055 (0.119)
7 or more cups a week	74 (56.5%)	84 (42.2%)	
Less than 7 cups a week	25 (19.1%)	53 (26.6%)	
None	31 (23.7%)	56 (28.1%)	
Missing	1 (0.8%)	6 (3.0%)	
Smoke			0.316 (0.476)
No	125 (95.4%)	182 (91.5%)	
Yes	5 (3.8%)	14 (7.0%)	
Missing	1 (0.8%)	3 (1.5%)	
Eats fruits or vegetables daily			0.028 (0.151)
No	15 (11.5%)	42 (21.1%)	
Yes	115 (87.8%)	152 (76.4%)	
Missing	1 (0.8%)	5 (2.5%)	
Eats grains daily			0.787 (0.779)
No	42 (32.1%)	59 (29.6%)	
Yes	86 (65.6%)	133 (66.8%)	
Missing	3 (2.3%)	7 (3.5%)	
Eats meats daily			0.403 (0.418)
No	49 (37.4%)	83 (41.7%)	
Yes	81 (61.8%)	110 (55.3%)	
Missing	1 (0.8%)	6 (3.0%)	
Other neuro disease			0.252 (0.137)
No	122 (93.1%)	172 (86.4%)	
Yes	8 (6.1%)	5 (2.5%)	
Missing	1 (0.8%)	22 (11.1%)	
Cancer			0.192 (0.080)
No	119 (90.8%)	183 (92.0%)	
Yes	8 (6.1%)	5 (2.5%)	
Missing	4 (3.1%)	11 (5.5%)	
Underweight (BMI < 18.5)			0.239 (0.011)
Yes	2 (1.5%)	9 (4.5%)	
No	125 (95.4%)	183 (92.0%)	
Missing	4 (3.1%)	7 (3.5%)	

Logistic regression with PD as dependent variable, with age and sex as confounders was performed, and adjusted *p*-value was calculated using F-test between full model and reduced model.

*SD* standard deviation.

**Table 2:** Comparing Parkinson’s disease patients with and without underweight on demographic variables.

	Underweight (*N* = 9)	Non-underweight (*N* = 183)	*P*-value (adjusted *P*-value)
Age			0.316
Mean (SD)	71.8 (7.79)	68.1 (9.18)	
Sex			0.01
Female	7 (77.8%)	56 (30.6%)	
Male	2 (22.2%)	127 (69.4%)	
Depression			0.296 (0.119)
No	8 (88.9%)	116 (63.4%)	
Yes	1 (11.1%)	59 (32.2%)	
Missing	0 (0%)	8 (4.4%)	
Anxiety			0.469 (0.194)
No	8 (88.9%)	129 (70.5%)	
Yes	1 (11.1%)	50 (27.3%)	
Missing	0 (0%)	4 (2.2%)	
Inflammatory bowel disease			1 (0.995)
No	8 (88.9%)	164 (89.6%)	
Yes	1 (11.1%)	16 (8.7%)	
Missing	0 (0%)	3 (1.6%)	
Irritable bowel syndrome			0.153 (0.201)
No	5 (55.6%)	168 (91.8%)	
Yes	2 (22.2%)	12 (6.6%)	
Missing	2 (22.2%)	3 (1.6%)	
Constipation			0.446 (0.133)
No	9 (100%)	154 (84.2%)	
Yes	0 (0%)	27 (14.8%)	
Missing	0 (0%)	2 (1.1%)	
Gastrointestinal symptoms other than constipation			0.675 (0.495)
No	5 (55.6%)	72 (39.3%)	
Yes	4 (44.4%)	97 (53.0%)	
Missing	0 (0%)	14 (7.7%)	
Eats fruits or vegetables daily			0.842 (0.742)
No	1 (11.1%)	40 (21.9%)	
Yes	7 (77.8%)	142 (77.6%)	
Missing	1 (11.1%)	1 (0.5%)	
Eats grains daily			0.431 (0.181)
No	1 (11.1%)	58 (31.7%)	
Yes	7 (77.8%)	122 (66.7%)	
Missing	1 (11.1%)	3 (1.6%)	
Eats meats daily			0.992 (0.819)
No	3 (33.3%)	80 (43.7%)	
Yes	5 (55.6%)	101 (55.2%)	
Missing	1 (11.1%)	2 (1.1%)	
Eats yogurt daily			0.610 (0.506)
Less than once a month or never	2 (22.2%)	69 (37.7%)	
Few times a month	3 (33.3%)	45 (24.6%)	
Few times a week	3 (33.3%)	49 (26.8%)	
At least once a day	0 (0%)	16 (8.7%)	
Missing	1 (11.1%)	4 (2.2%)	
Probiotics			0.694
No	94 (71.8%)	141 (70.9%)	
Yes	33 (25.2%)	43 (21.6%)	
Missing	4 (3.1%)	15 (7.5%)	

Logistic regression with underweight status as dependent variable, with age and sex as confounders was performed, and adjusted *p*-value was calculated using F-test between full model and reduced model.

*SD* standard deviation.

### Gut microbiome structure and abundance differ significantly between PD underweight and non-underweight individuals

To investigate whether underweight in the PD group was associated with changes in the gut microbiota community structure, overall taxonomic alpha and beta diversities between underweight and non-underweight individuals in the PD group were compared, and the results are shown in Supplementary Fig. 2. Regarding alpha diversity, none of the four metrics (Observed, Chao1, Shannon index, Simpson index) was found to be significantly different between underweight and non-underweight individuals (pObserved = 0.8461; pChao1 = 0.7567; pShannon = 0.7684; pSimpson = 0.8891). In contrast, two of the three metrics of beta diversity (unweighted Unique Fraction distance (Unifrac) and Canberra distance) showed significant changes in community structure between underweight and non-underweight individuals in the PD group (pUnweighted-unifrac = 0.0376; pCanberra = 0.0353; pWeighted-unifrac = 0.5547).

**Fig. 1:**
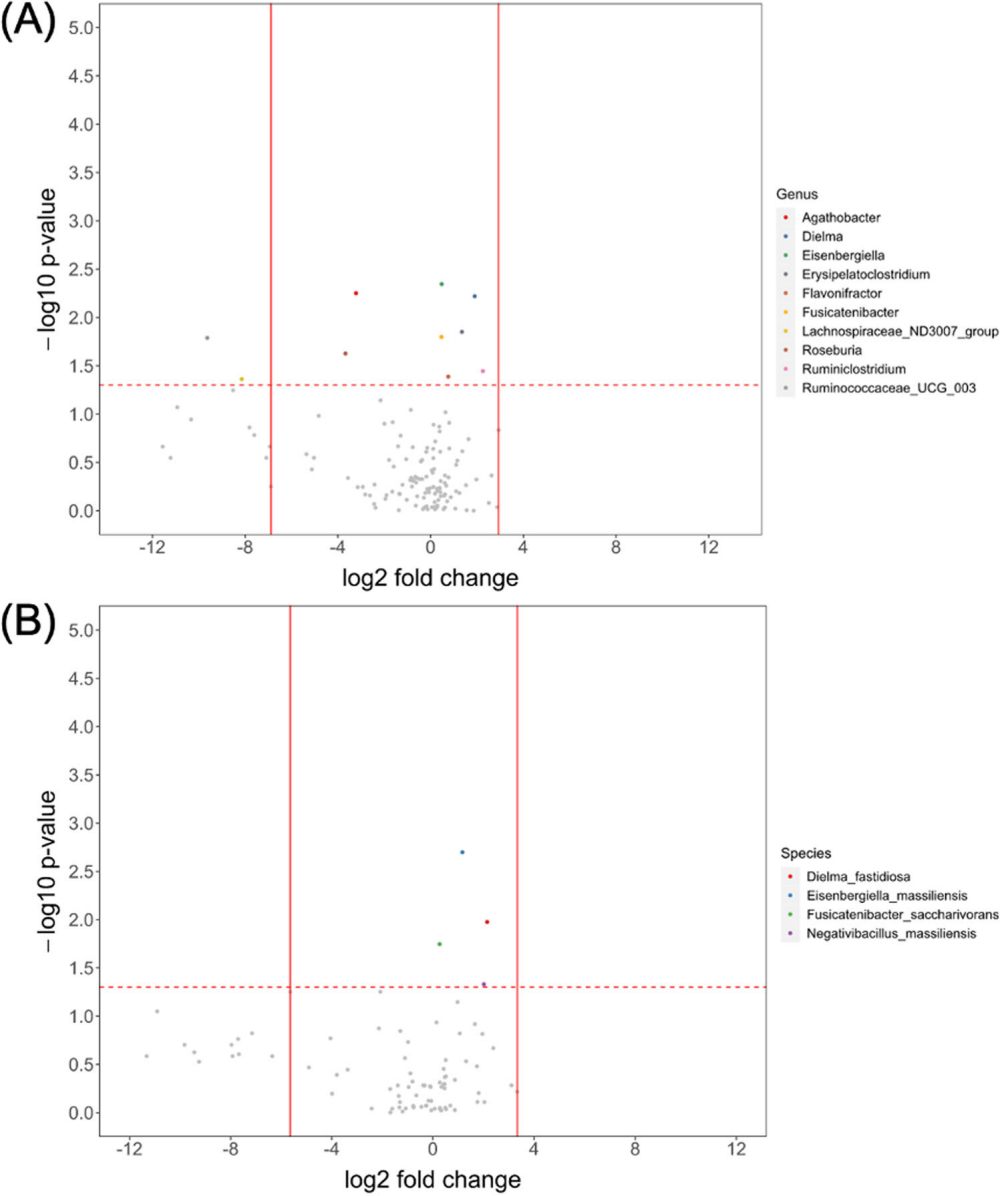
Microbial difference between underweight and non-underweight individuals in Parkinson’s disease group. **A** Genus. **B** Species. x-axis: fold change in relative abundance (log2 of underweight/non-underweight). y-axis: statistical significance (-log10 of *p* value). Points above dash line: *p* < 0.05.

**Fig. 2:**
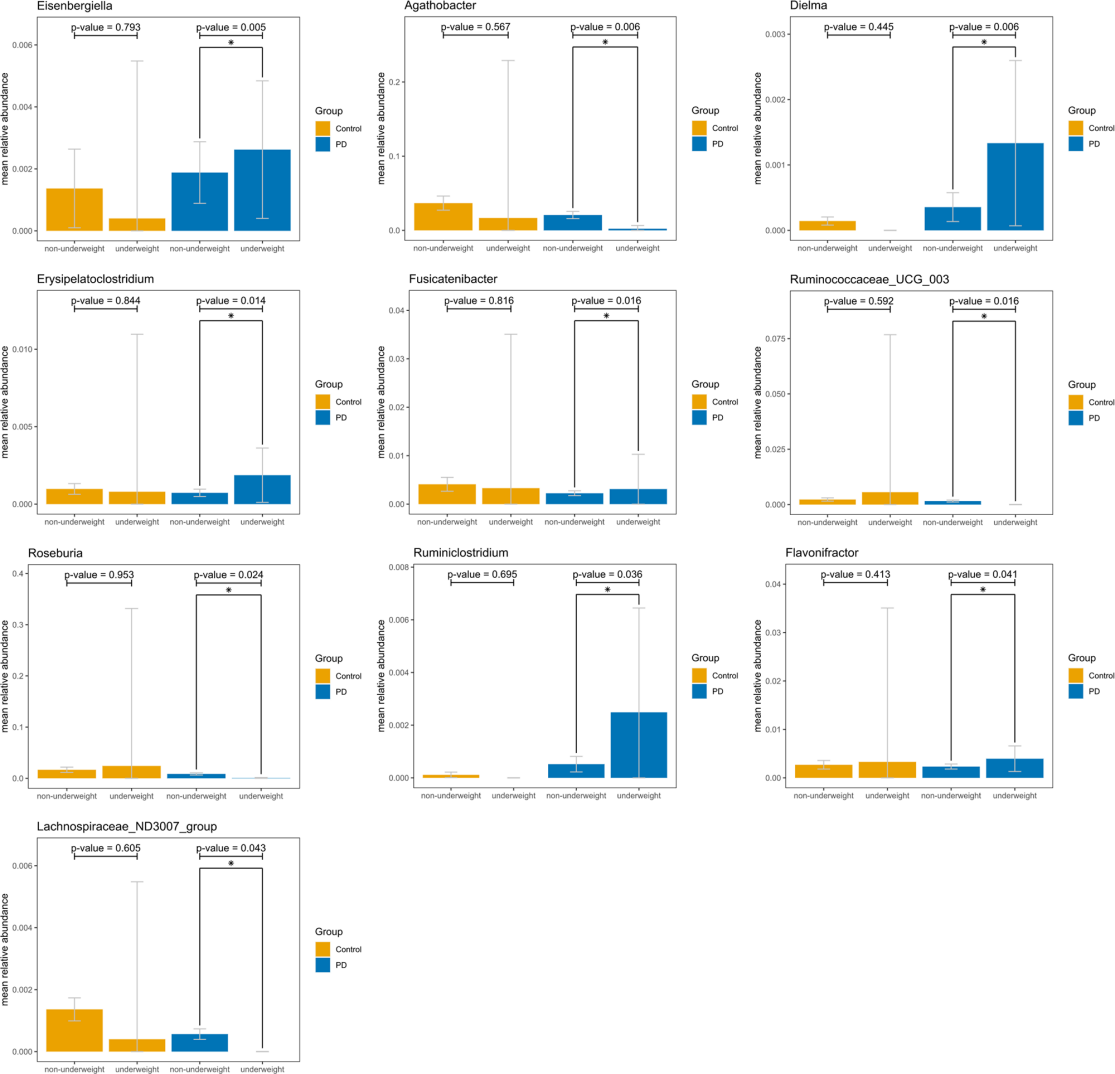
Relative abundance of the ten underweight-related genera in the Parkinson’s disease (PD) group. This assessment encompasses both underweight and non-underweight individuals within both the PD and control groups. error bars: mean +/- standard deviation.

To identify key intestinal bacteria associated with underweight in the PD group, comparison of microbial differences in relative abundance between underweight and non-underweight individuals in the PD group was performed, and the results are shown in Fig. 1, at the genus (Fig. 1A) and species levels (Fig. 1B). Ten genera and four species were significantly different in relative abundance between underweight and non-underweight individuals in PD group, with Ruminiclostridium, Dielma, Erysipelatoclostridium, Flavonifractor, Eisenbergiella and Fusicatenibacter showed increased abundance, and Ruminococcaceae_UCG_003, Lachnospiraceae_ND3007_group, Roseburia and Agathobacter showed decreased abundance. The same comparison was performed in the control group, and the results are shown in Supplementary Fig. 3, with four genera and four species significantly different in relative abundance. None of the ten underweight-related genera in the PD group showed an association with underweight in the control group (Fig. 2).

### Key intestinal bacteria are identified through mediation analysis

To identify the key intestinal bacteria that mediated the effect of PD on underweight, we performed mediation analysis, the results of which are shown in Table 3. Our results showed that the proportion mediated by intestinal microbial alterations (IMAs) was 42.29% at the genus level (Tables 3A) and 37.91% at the species level (Table 3B). Five genera showed significant mediation effects: Agathobacter (18.20%, *p* = 0.0016), Eisenbergiella (10.71%, *p* = 0.0162), Fusicatenibacter (19.10%, *p* = 0.0269), Roseburia (9.44%, *p* = 0.0377), and Ruminococcaceae_UCG_013 (5.82%, *p* = 0.0451). PD had a positive effect on the abundance of Eisenbergiella (1.0614), and the abundance of Eisenbergiella (0.1966) had a positive effect on underweight individuals. In contrast, PD had negative effects on the other four genera (Agathobacter: -2.1187, Fusicatenibacter: -1.6858, Roseburia: -1.5329, Ruminococcaceae_UCG_013: -0.9022), and these four genera had negative effects (Agathobacter: -0.2151, Fusicatenibacter: -0.1959, Roseburia: -0.1520, Ruminococcaceae_UCG_013: -0.1739). Two species, Fusicatenibacter saccharivorans (20.10%, *p* = 0.0076) and Roseburia inulinivorans (13.17%, *p* = 0.0237), had significant mediation effects. The aforementioned bacteria were included in the significant taxa presented in Fig. 1.

**Table 3:** Mediation analysis for the mechanism of PD-related underweight mediated by intestinal microbial alteration with different measures as mediators: (A) genus level; and (B) species level.

(A)					
	Effect of PD on underweight (95% CI)	Effect of PD on microbiota (95% CI)	Effect of microbiota on underweight (95% CI)	PM (%)	*P*-value
Unadjusted	1.8079 (0.3013, 3.3145)				
Adjusted for					
All genera	1.0433			42.29 %	
* Agathobacter*	1.4788 (0.1098, 2.8478)	−2.1187 (−3.0017, −1.2357)	−0.2151 (−0.3478, −0.0824)	18.20 %	0.0016
*Eisenbergiella*	1.6143 (0.3160, 2.9126)	1.0614 (0.2804, 1.8424)	0.1966 (0.0370, 0.3561)	10.71 %	0.0162
*Fusicatenibacter*	1.4626 (0.0136, 2.9116)	−1.6858 (−2.4641, −0.9075)	−0.1959 (−0.3688, −0.0230)	19.10 %	0.0269
* Roseburia*	1.6373 (0.2010, 3.0736)	−1.5329 (−2.4245, −0.6413)	−0.152 (−0.2947, −0.0093)	9.44 %	0.0377
*Ruminococcaceae_UCG_013*	1.7027 (0.3834, 3.0220)	−0.9022 (−1.6531, −0.1513)	−0.1739 (−0.3434, −0.0044)	5.82 %	0.0451

(B)					
	Effect of PD on underweight (95% CI)	Effect of PD on microbiota (95% CI)	Effect of microbiota on underweight (95% CI)	PM (%)	*P*-value
Unadjusted	1.8079 (0.3013, 3.3145)				
Adjusted for					
All species	1.1224			37.91 %	
*Fusicatenibacter_saccharivorans*	1.4446 (0.0487, 2.8405)	−1.6595 (−2.4485, −0.8704)	−0.2490 (−0.4304, −0.0675)	20.10 %	0.0076
*Roseburia_inulinivorans*	1.5698 (0.2537, 2.8859)	−1.7441 (−2.6531, −0.8351)	−0.2935 (−0.5465, −0.0405)	13.17 %	0.0237

*PM* proportion mediated, *CI* confident interval.

## Discussion

Unintentional weight loss is an important clinical problem in PD patients. Our results showed that PD patients tend to have a lower BMI and are more likely to develop underweight than controls. A previous study showed that PD patients frequently experience sarcopenia, with an estimated prevalence of 6% to 55.5%^27^ and malnutrition, with an estimated prevalence of 0% to 24%^28^. The combination of weight loss, muscle wasting, and decreased appetite observed in patients with PD is commonly referred to as PD-related cachexia^29,30^. This condition is associated with a poorer clinical prognosis, including impaired physical function, reduced quality of life, longer hospital stays, and increased mortality^31^. However, the mechanism underlying underweight in PD remains largely unknown. Therefore, investigating the mechanism of underweight in PD and providing appropriate interventions are important issues.

Several mechanisms have been proposed to explain the occurrence of underweight in patients with PD. Tremor and rigidity (increased muscular tone and resistance) are the core motor symptoms of PD, which may result in increased energy expenditure and, therefore, decreased body weight^32^. Non-motor symptoms, such as olfactory dysfunction, neuropsychiatric symptoms (depression, anxiety), and gastrointestinal dysfunction, have been linked to weight loss in PD patients due to decreased food intake^33,34^. PD patients with anosmia had been shown to have a higher frequency of weight loss than those without anosmia^35^. Depression has been associated with weight loss or malnutrition in PD patients^36,37^. Constipation had been reported to be one of risk factors of malnutrition in community dwelling people with PD^38^. In addition to clinical symptoms, perturbation of hypothalamic metabolic regulation has also been proposed to be involved in the mechanism of underweight PD. The hypothalamus receives and integrates orexigenic and anorexigenic signals to regulate appetite and food intake, which are modulated by dopaminergic and serotonergic systems in the brain and peripheral neuroendocrine signaling, such as ghrelin and leptin^39^. Neurodegeneration of the serotonergic system with low levels of serotonin has been described in PD, and loss of serotonergic neurons has been shown to result in a decrease in body weight in a PD rat model^40^. Patients with PD experiencing weight loss have been shown to have lower plasma leptin and ghrelin levels than those without weight loss^41^. Dysregulation of these systems may result in changes in eating behavior and, therefore, decreased body weight^39^. Although there are many hypotheses regarding PD-related underweight, our results show that there are no significant differences in depression, anxiety, gastrointestinal symptoms, and diet habits between underweight and non-underweight individuals with PD. These findings may indicate that there are other factors involved in the underlying mechanism of underweight in PD, and gut microbiota may play a key role.

Numerous studies have demonstrated alterations in the gut microbiota composition in PD^42^, prompting our investigation of the potential role of gut microbiota in the mechanism of underweight in PD. Our analysis revealed significant inter-individual variation in the gut microbiota of underweight PD patients compared to non-underweight PD patients. A similar finding was reported in anorexia nervosa patients, with increased inter-individual variation in the anorexia nervosa group compared with controls^43^. Comparison of the genus-level microbiota composition between underweight and non-underweight individuals in the PD group revealed significant differences in the abundance of 10 genera. Notably, five of these genera are known to be involved in the production of SCFAs, which were mostly decreased in underweight individuals in the PD group. Similar changes in the gut microbiota profile have been described in anorexia nervosa patients, with increased *Eisenbergiella* and decreased *Lachnospiraceae*, *Agathobacter*, *Ruminococcaceae* and *Roseburia*^43–46^. *Fusicatenibacter*, one of the SCFA-producing bacteria, was found to be a predictive marker for the progression of early PD, with an area under the receiver operating characteristic curve as high as 0.861 in random forest models for predicting the progression of Hoehn and Yahr stages over a period of two years^47^. Reduced *Fusicatenibacter* and *Ruminococcaceae* were also shown in PD patients with deteriorated Hoehn and Yahr stages in the following two years^47^. In a separate study, *Lachnospiraceae* were found to be associated with gait speed and physical frailty^48^. The severity and progression of PD are strongly related to underweight^13^, which may explain why the bacteria mentioned above are associated with underweight in PD. In addition, our analysis showed that the ten underweight-related genera in the PD group did not show a similar association in the control group. This suggests that these genera are specifically associated with underweight in the PD population, but not in the general population.

To identify which of the ten underweight-related genera in PD were involved in the mechanism of underweight in PD, mediation analysis was performed, with gut microbiota as the mediator between PD and underweight. We found that the 42.29% effect of PD on underweight was mediated by gut microbiota, and one pro-inflammatory genus, *Eisenbergiella*, and four SCFA-producing genera, *Fusicatenibacter*, *Agathobacter*, *Roseburia* and *Ruminococcaceae_UCG_003*, had significant mediation effects, indicating the importance of gut microbiota in underweight PD. Increased pro-inflammatory genera along with decreased SCFA-producing genera may be involved in the underweight mechanism in PD. Previous studies have reported that similar changes in the gut microbiota profile are predictive of the accelerated progression of PD^47^.

Increased levels of pro-inflammatory cytokines have been found in patients^49^. Additionally, pro-inflammatory cytokines can activate a series of molecular pathways involved in skeletal muscle wasting^50,51^. High levels of inflammatory cytokines had been demonstrated to be negatively related to muscle strength and mass^52,53^. *Eisenbergiella*, a newly isolated anaerobic bacterial strain, can produce succinate as a metabolic end products^54^. Succinate can serve as a proinflammatory signal in the immune system. It induces the differentiation of T lymphocytes into pro-inflammatory TH17 cells^55^ and enhances the production of pro-inflammatory cytokines (TNFα and Il-1β) in dendritic cells^56,57^. SCFAs, including acetate, butyrate, and propionate, are produced by the bacterial fermentation of non-digestible carbohydrates containing fewer than six carbons and serve as major energy sources for colonic cells^58^. Decreased SCFAs levels in the gut can lead to disruption of the intestinal barrier and increased intestinal permeability, which facilitates the translocation of toxic bacterial products into the systemic circulation, and therefore, the development of an inflammatory state^59,60^. Acetate, butyrate, and propionate can also regulate intestinal inflammation by suppressing pro-inflammatory cytokine production^61–63^. Taken together, our findings suggest that the pro-inflammatory state induced by an increase in pro-inflammatory genera and a decrease in SCFA-producing genera in the gut may play a role in the mechanism of underweight and sarcopenia in PD.

SCFAs have been reported to affect skeletal muscle metabolism by increasing fatty acid oxidation, preventing lipid accumulation, increasing glycogenesis, and inhibiting glycolysis^64^. After being produced by bacteria in the gut, SCFAs enter the portal vein, followed by systemic circulation, where they act as signaling molecules in the skeletal muscle. These SCFA-mediated signalling pathways in skeletal muscles are involved in a range of physiological processes, such as triggering the release of glucagon-like peptide-1, leptin, and insulin in colons^65,66^ and^67^, respectively. SCFAs supplementation has been shown to have potential benefits in preventing aging-related muscle atrophy in mice^68^. Furthermore, studies have found that germ-free mice lacking gut microbiota have increased skeletal muscle mass after administration of SCFAs supplements^69^. In addition, SCFAs have been found to modulate insulin signalling, adipogenesis, and lipolysis in adipocytes^58^. This finding may be relevant to recent research that showed that PD patients have a greater loss of visceral and subcutaneous fat, but not muscle, compared to controls^70^. SCFAs can also induce neuronal activation in the peripheral nervous system of the gut (enteric nervous system and vagal afferents), which results in increased activity of the dorsal vagal complex^71,72^. The dorsal vagal complex receives inputs from the vagus nerve and hypothalamus, a key brain region involved in appetite and metabolism control. Taken together, disturbances in metabolism and appetite regulation induced by decreased SCFA-producing genera in the gut may be involved in the mechanism of underweight in PD.

This study had several limitations. First, while assessing the severity of underweight and discussing the proposed mechanism of underweight PD, several variables were not measured, such as the body weight change, body muscle mass, severity of motor symptoms, symptoms of olfactory dysfunction, blood and stool laboratory data for inflammation markers and hormones, and amount of daily intake, which limits the comparison with previous studies discussing underweight PD. Second, while we propose that SCFAs play a significant role in the mechanism of underweight PD, there is a lack of direct measurement of SCFAs levels in blood or stool. Third, 16S rRNA sequence data was used in this study for bacterial identification. 16S rRNA sequencing technology is known for having a low resolution at species level in terms of bacterial identification. There was a lot of “NA” in species-level annotation, which strongly affected the mediation effects being detected at species level. We would like to incorporate shotgun metagenomic sequencing data into our future studies in order to achieve results with higher accuracy at measuring the effect contributed by species level. Fourth, for a valid mediation analysis, all the confounding factors between PD and underweight (UW), PD and IMA, and IMA and UW should be controlled. Age and sex were confounding factors of PD-UW, PD-IMA, and IMA-UW relationship, which had been controlled in this study. However, there are also many potential confounding factors of PD-IMA relationship (e.g., occupational and environmental exposure^73,74^) and IMA-UW relationship (e.g., socioeconomic status and dietary patterns^75,76^) not being measured in this study. We will incorporate them into our future studies. This will help with predicting the unbiased mediation effect contributed by gut microbiota in PD underweight. Finally, only 42.29% of the effect of PD on underweight is explained by the gut microbiota, indicating that a large proportion of underweight PD is still unknown. Therefore, further studies are needed to investigate the underlying mechanisms of PD.

This study was a pilot study for investigating the mediation effect of gut microbiota on PD underweight. The next step of this pilot study will be collecting longitudinal data with regularly assessed body weight, fecal samples, and Unified PD Rating Scale score, to ensure the causal mediation effect of gut microbiota on PD underweight and assess its clinical impact. More potential confounding factors such as occupational and environmental exposure, socioeconomic status, and dietary patterns will also be collected and controlled in our future work since randomized trial cannot be performed. We would also like to analyse shotgun metagenomic sequencing data for a higher resolution of bacteria at species level, which can more accurately predict the mediation effect at species level.

In conclusion, we found that up to 42.29% of the effect of underweight PD is mediated through gut microbiota, with increased pro-inflammatory bacteria and decreased SCFA-producing bacteria. Our results indicate that the pro-inflammatory state, disturbance of metabolism, especially in the muscle, and interference of appetite regulation may be involved in the mechanism of PD-related underweight. In addition, SCFAs supplements and SCFA-producing probiotics may hold promise for managing underweight individuals with PD.

## Methods

### Participant recruitment and data collection

Our data were adapted from the study of Hill‐Burns et al. ^77^, in which 330 participants (185 male, 145 female; mean age 69.2) were enrolled from the NeuroGenetics Research Consortium from 2014/3 to 2015/1. The methods and clinical and genetic characteristics of the NeuroGenetics Research Consortium dataset were described in detail by Hamza et al. ^78^. Among the 330 participants, 199 (133 male, 66 female; mean age 68.4) were diagnosed with PD using the modified UK Brain Bank criteria. The remaining 131 controls (52 male and 79 female; mean age 70.4) were self-reporting free of neurodegenerative disease. Underweight was defined as a BMI of <18.5^79^. There were 9 underweight cases in the PD group and 2 cases in the control group. Details of the fecal sample collection process, DNA extraction and sequencing, and metadata collection can be found in Hill-Burns et al.^77^. All data has been used in Hill-Burns et al.^77^, therefore no ethical statements is required.

### Processing of 16S rRNA sequence data

The 16S rRNA gene is highly conserved among the bacteria. Therefore, it is highly suitable as a target gene for DNA sequencing for bacterial identification. Sequence reads were processed using Trimmomatic v0.39^80^ to remove adaptors. The outputs were then processed, aligned, and categorized using DADA2 1.16^81^. Briefly, sequence reads were first filtered using DADA2’s recommended parameters. Filtered reads were then de-replicated and de-noised using the DADA2 default parameters. After building the amplicon sequence variant (ASV) table and chimeras were removed, taxonomy was assigned using SILVA v132, natively implemented in DADA2. We used the addSpecies function in DADA2 to add species-level annotation, with SILVA as a reference. Sequence counts were normalized to relative abundance (calculated by dividing the number of sequences assigned to a unique ASV by the total sequence count in the sample). Bacteria that were present in more than 10% of the samples were used in subsequent analyses.

### Statistical analyses

We compared demographic characteristics (including age, sex, race, BMI, residence location, alcohol consumption amount, coffee consumption amount, smoking status, diet habits, other neurological diseases, presence of cancer, and underweight) between the PD and control groups using the Wilcoxon test for continuous variables and chi-square test for categorical variables. Logistic regression with PD as the dependent variable, with age and sex as confounders, was performed, and the adjusted *p*-value was calculated using the F-test between the full model and the reduced model (without the target independent variable). We also compared age, sex, depression, anxiety, inflammatory bowel disease, irritable bowel disease, constipation, gastrointestinal symptoms other than constipation, and diet habits (including eating fruits or vegetables daily, eating grains daily, eating meats daily, eating yogurt daily and probiotics intake) between underweight and non-underweight individuals in the PD group using the Wilcoxon test for continuous variables and the Chi-square test for categorical variables. The adjusted *p* value was also calculated as stated above, except for underweight status as the dependent variable.

We compared the overall taxonomic diversity between underweight and non-underweight individuals in the PD group by calculating alpha and beta diversities, which incorporate both species richness and evenness. Regarding alpha diversity, we estimated the observed richness (i.e., number of ASVs), Chao1, Shannon, and Simpson indices from the ASV table^82–84^ using Phyloseq 1.32.0^85^. *P*-values for alpha diversity were calculated by ANOVA using Stats 4.0.5. Regarding beta diversity, we estimated the dissimilarities (distances) between the two groups using the following metrics: unweighted unique fraction metrics (Unifrac), weighted Unifrac^86^, and Canberra distance^87^. Beta diversity indices for weighted and unweighted UniFrac were calculated using Phyloseq 1.32.0^85^. The Canberra distance was calculated using Vegan 2.5.7. *P*-values for beta diversity were calculated with ADONIS using vegan 2.5.7. We also compared the microbial differences in relative abundance between underweight and non-underweight individuals separately for both the PD and control groups using the Wilcoxon test. The relative abundance of gut microbiota, which was significantly different in relative abundance between underweight and non-underweight individuals in the PD group, was calculated among underweight and non-underweight individuals in both the PD and control groups.

### Mediation analysis

The mechanism of PD-related underweight is primarily mediated by microbiome alterations. Mediation analysis was employed to measure the degree to which the IMA between individuals with PD and controls explains the causal relationship between PD and underweight, and to identify key bacterial taxa that play a significant role in mediating the relationship between PD and underweight. PD status was the exposure variable, underweight status was the outcome of interest, and IMA was the mediator. Sex and age were adjusted for mediation analysis. The directed acyclic graph of mediation analysis is shown in Supplementary Fig. 1.

Three statistical models were constructed. In Model 1, we built a regular logistic regression with underweight as the dependent variable while PD and baseline confounders as independent variables. In Model 2, we built another logistic regression with underweight as the dependent variable while PD, gut microbial alteration, and baseline confounders as independent variables. Because microbial alteration is a high dimensional variable, we adapted a quasi-binomial logistic regression algorithm. In Model 3, we built a linear regression model with gut microbial alteration as a dependent variable while PD and baseline confounders as independent variables. Here the coefficient of PD in Models 1 and 2 was interpreted as the total effect of PD on underweight and the direct effect (the effect of PD on underweight that is not mediated through IMA), respectively. The coefficient of PD in Model 3 represented the effect of PD on microbial alteration, and the coefficient of gut microbial alteration in Model 2 represented the effect of each measurement of IMA on underweight. The mediation effect (the effect of PD on underweight that is mediated through any IMA) was measured by the difference of the coefficients of PD between Model 1 and 2 and proportion mediated was calculated as mediation effect divided by total effect. Joint hypothesis tests were used for calculating *p*-values of the mediation effect. All statistical analyses were performed with R version 3.6.0., under which Ridge regression analysis was performed using glmnet (version 4.1-2) and linear model and logistic regression were built and performed using stats (version 4.1.0).

### Reporting summary

Further information on research design is available in the Nature Research Reporting Summary linked to this article.

### Supplementary information

Supplementary Information of “Unravelling the mechanisms of underweight in Parkinson’s Disease by investigating into the role of gut microbiome”

Reporting Summary

## Data Availability

The sequences analyzed in this study are accessible at the European Nucleotide Archive (ENA) under accession number ERP016332.

## Code availability

Researchers interested in access to the code may contact Y.-L.C. at yan9914@nycu.edu.tw.
