# Supplementary material for: Unravelling the mechanisms of underweight in Parkinson’s disease by investigating into the role of gut microbiome
Source: NPJ Parkinsons Dis. 2024 Jan 24;10:28. doi: 10.1038/s41531-023-00587-w (PMC10808448; doi:10.1038/s41531-023-00587-w)
Supplement: Supplementary file 1 — Supplementary Information of “Unravelling the mechanisms of underweight in Parkinson’s Disease by investigating into the role of gut microbiome” [file 41531_2023_587_MOESM1_ESM.pdf]

Supplementary Information of "Unravelling the mechanisms of underweight in Parkinson's Disease by investigating into the role of gut microbiome"

Supplementary Methods

We have also conducted another set of mediation analysis with the Tobit model [1] to replace model 3. The results are presented in Supplementary Tables 1. It was implemented with the R package "crch" [2,3]. The Tobit model has milder assumptions compared to conventional linear regression, enabling analysis under zero-inflation conditions and the consideration of conditional heteroscedasticity. This may avoid the problem of misspecification that can occur with traditional linear models. Compared to Table 3 in the main text, the utilization of the Tobit model to estimate the effect of PD on gut microbial alteration does not alter our conclusions.

Supplementary Figure 1. Directed acyclic graph of mediation analysis. PD status is the exposure variable, underweight status is the outcome of interest, and intestinal microbial alteration is the mediator. Sex and age are confounders.

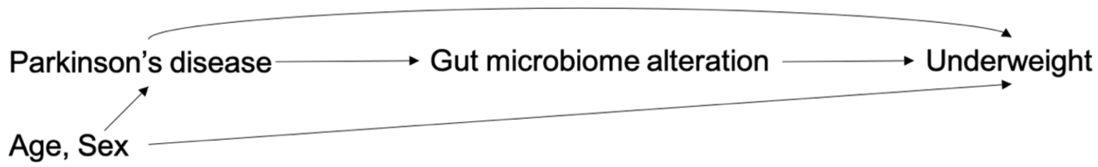

Supplementary Figure 2. Difference in microbiota structure between underweight and non-underweight individuals in Parkinson's disease group. (A) Alpha diversity. (B) Beta diversity. In boxplot, median are shown as centre line, and 25 percentile and 75 percentile are shown as bounds of box. Whiskers are 1.5 times interquartile range away from bounds of box. BMI: body mass index.

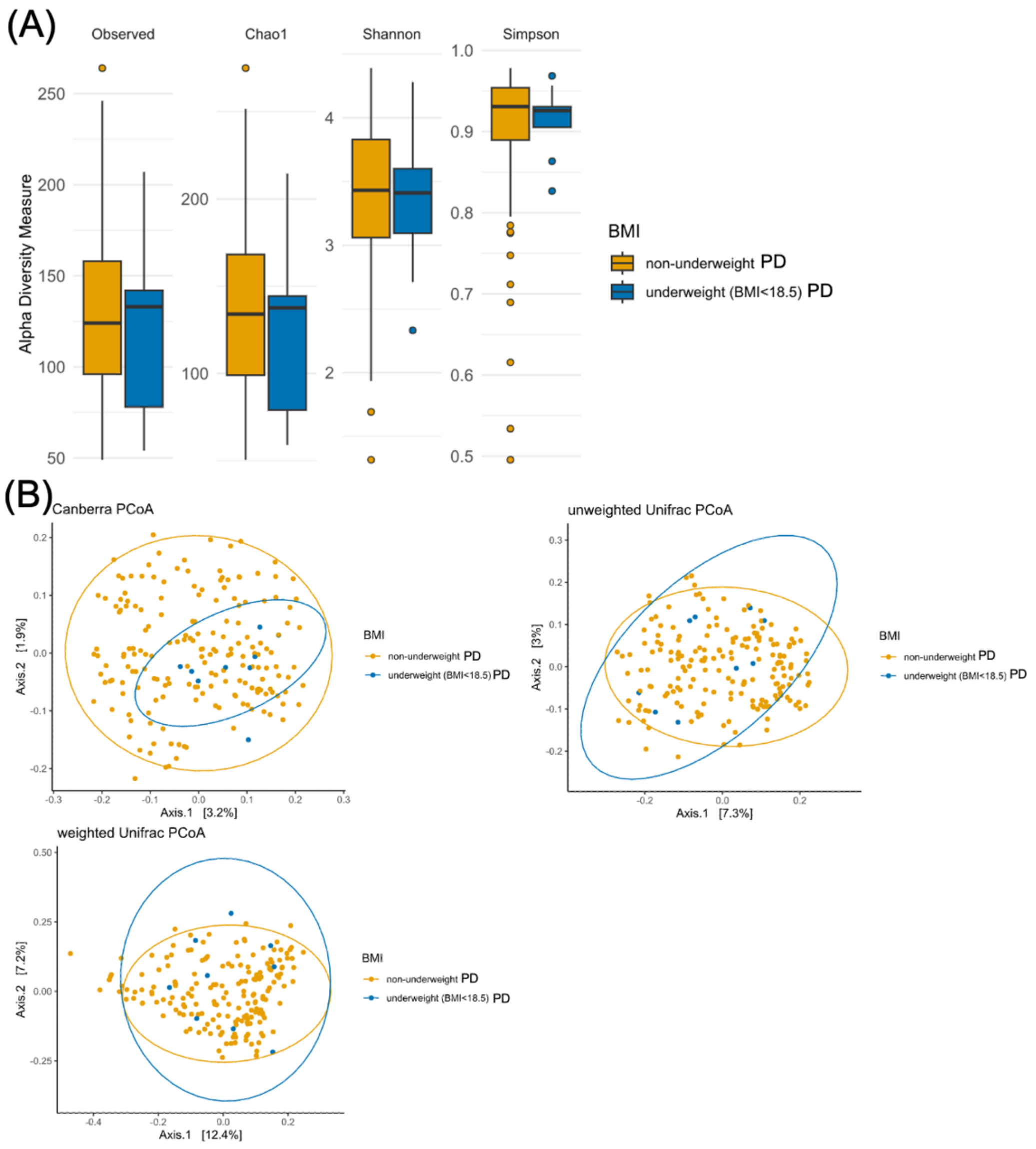

Supplementary Figure 3. Microbial difference between underweight and non-underweight individuals in control group. (A) Genus. (B) Species. x-axis: fold change in relative abundance (log2 of underweight / non-underweight). y-axis: statistical significance (-log10 of p value). Points above dash line: $p < 0.05$

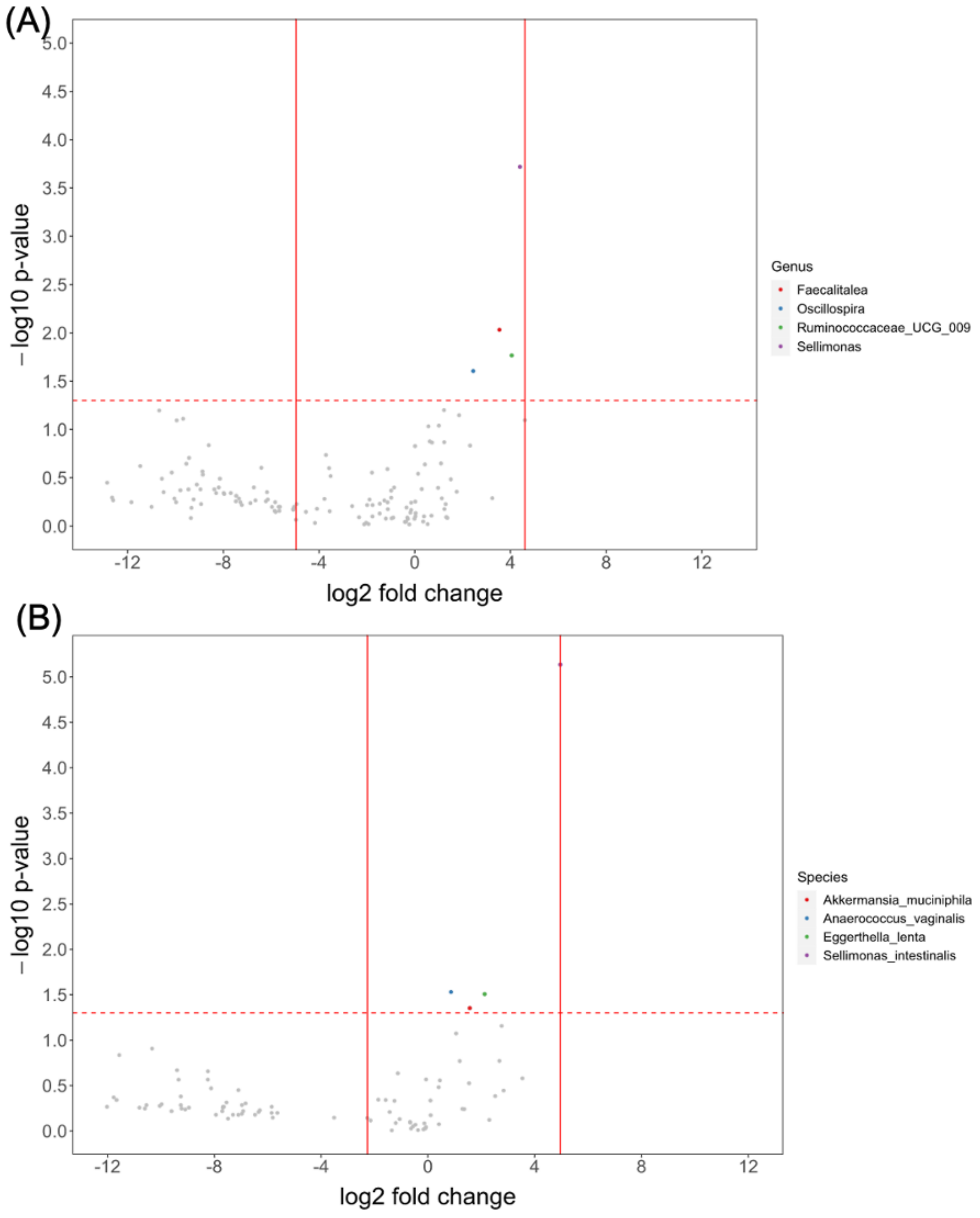

Supplementary Table 1. Mediation analysis (using the Tobit model) investigating the mechanism of PD-related underweight, mediated by intestinal microbial alteration, with various measures as mediators: (A) genus level; and (B) species level

(A)

	Effect of PD on underweight (95% CI)	Effect of PD on microbiota (95% CI)	Effect of microbiota on underweight (95% CI)	PM (%)	P-value
Unadjusted	1.8079				
	(0.3013, 3.3145)				
Adjusted for					
All genera	1.0433			42.29 %	
Agathobacter	1.4788	-2.8762	-0.2151	18.20 %	0.0016
	(0.1098, 2.8478)	(-3.9037, -1.8487)	(-0.3478, -0.0824)		
Eisenbergiella	1.6143	3.2468	0.1966	10.71 %	0.0162
	(0.3160, 2.9126)	(0.835 ,5.6587)	(0.0370, 0.3561)		
Fusicatenibacter	1.4626	-2.7700	-0.1959	19.10 %	0.0269
	(0.0136, 2.9116)	(-3.8647, -1.6753)	(-0.3688, -0.0230)		
Roseburia	1.6373	-2.3744	-0.152	9.44 %	0.0377
	(0.2010, 3.0736)	(-3.5893, -1.1596)	(-0.2947, -0.0093)		
Ruminococcaceae_UCG_013	1.7027	-1.5542	-0.1739	5.82 %	0.0451
	(0.3834, 3.0220)	(-2.7056, -0.4029)	(-0.3434, -0.0044)		

(B)

	Effect of PD on underweight	Effect of PD on microbiota	Effect of microbiota on	PM (%)	P-value

	(95% CI)	(95% CI)	underweight (95% CI)		
Unadjusted	1.8079				
	(0.3013, 3.3145)				
Adjusted for					
All species	1.1224			37.91 %	
Fusicatenibacter_saccharivorans	1.4446	-2.7909	-0.2490	20.10 %	0.0076
	(0.0487, 2.8405)	(-3.9307, -1.6512)	(-0.4304, -0.0675)		
Roseburia_inulinivorans	1.5698	-4.5341	-0.2935	13.17 %	0.0237
	(0.2537, 2.8859)	(-6.8049, -2.2632)	(-0.5465, -0.0405)		

PM: Proportion mediated; CI: Confident Interval

Supplementary References

1. Tobin, J. (1958). Estimation of relationships for limited dependent variables. *Econometrica: journal of the Econometric Society*, 24-36.
2. Messner, J. W., Zeileis, A., Broecker, J., & Mayr, G. J. (2014). Probabilistic wind power forecasts with an inverse power curve transformation and censored regression. *Wind Energy*, *17*(11), 1753-1766.
3. Messner, J. W., Mayr, G. J., & Zeileis, A. (2016). Heteroscedastic Censored and Truncated Regression with crch. *R J.*, *8*(1), 173.
