# Supplementary material for: Unravelling the mechanisms of underweight in Parkinson’s disease by investigating into the role of gut microbiome
Source: NPJ Parkinsons Dis. 2024 Jan 24;10:28. doi: 10.1038/s41531-023-00587-w (PMC10808448; doi:10.1038/s41531-023-00587-w)
Supplement: Supplementary file 2 — Reporting Summary [file 41531_2023_587_MOESM2_ESM.pdf]

nature portfolio

Corresponding author(s): Shih-Chen Fu

Last updated by author(s): Aug 28, 2023

Reporting Summary

Nature Portfolio wishes to improve the reproducibility of the work that we publish. This form provides structure for consistency and transparency in reporting. For further information on Nature Portfolio policies, see our Editorial Policies and the Editorial Policy Checklist.

Statistics

For all statistical analyses, confirm that the following items are present in the figure legend, table legend, main text, or Methods section.

n/a	Confirmed	
☐	☒	The exact sample size (*n*) for each experimental group/condition, given as a discrete number and unit of measurement
☐	☒	A statement on whether measurements were taken from distinct samples or whether the same sample was measured repeatedly
☐	☒	The statistical test(s) used AND whether they are one- or two-sided<br>*Only common tests should be described solely by name; describe more complex techniques in the Methods section.*
☐	☒	A description of all covariates tested
☐	☒	A description of any assumptions or corrections, such as tests of normality and adjustment for multiple comparisons
☐	☒	A full description of the statistical parameters including central tendency (e.g. means) or other basic estimates (e.g. regression coefficient) AND variation (e.g. standard deviation) or associated estimates of uncertainty (e.g. confidence intervals)
☐	☒	For null hypothesis testing, the test statistic (e.g. *F*, *t*, *r*) with confidence intervals, effect sizes, degrees of freedom and *P* value noted<br>*Give P values as exact values whenever suitable.*
☒	☐	For Bayesian analysis, information on the choice of priors and Markov chain Monte Carlo settings
☒	☐	For hierarchical and complex designs, identification of the appropriate level for tests and full reporting of outcomes
☐	☒	Estimates of effect sizes (e.g. Cohen's *d*, Pearson's *r*), indicating how they were calculated

Our web collection on statistics for biologists contains articles on many of the points above.

Software and code

Policy information about availability of computer code

Data collection	no software was used
Data analysis	Trimmomatic v0.39, DADA2 1.16, SILVA v132, Phyloseq 1.32.0, R version 3.6.0

For manuscripts utilizing custom algorithms or software that are central to the research but not yet described in published literature, software must be made available to editors and reviewers. We strongly encourage code deposition in a community repository (e.g. GitHub). See the Nature Portfolio guidelines for submitting code & software for further information.

Data

Policy information about availability of data

All manuscripts must include a data availability statement. This statement should provide the following information, where applicable:

- Accession codes, unique identifiers, or web links for publicly available datasets
- A description of any restrictions on data availability
- For clinical datasets or third party data, please ensure that the statement adheres to our policy

The sequences analyzed in this study are accessible at the European Nucleotide Archive (ENA) under accession number ERP016332. All data were open access and de-identified

Research involving human participants, their data, or biological material

Policy information about studies with human participants or human data. See also policy information about sex, gender (identity/presentation), and sexual orientation and race, ethnicity and racism.

Reporting on sex and gender	Our data were adapted from the study of Hill-Burns et al. All data are open access and de-identified. No ethical approval is required. In short, 330 participants (185 male, 145 female; mean age 69.2) were enrolled from the NeuroGenetics Research Consortium in Seattle, Washington; Atlanta, Georgia; and Albany, New York from 2014/3 to 2015/1. The methods and clinical and genetic characteristics of the NeuroGenetics Research Consortium dataset were described in detail by Hamza et al.
Reporting on race, ethnicity, or other socially relevant groupings	Our data were adapted from the study of Hill-Burns et al. All data are open access and de-identified. No ethical approval is required.
Population characteristics	Our data were adapted from the study of Hill-Burns et al. All data are open access and de-identified. In short, institutional review boards and human subject committees at participating institutions approved the study of Hill-Burns et al. Written informed consent was obtained. Among the 330 participants, 199 (133 men, 66 women, mean age 68.4) were diagnosed with PD by the modified UK Brain Bank criteria. The remaining 131 controls (52 men, 79 women, mean age 70.4) were self-reporting free of neurodegenerative disease.
Recruitment	Our data were adapted from the study of Hill-Burns et al. All data are open access and de-identified. No ethical approval is required.
Ethics oversight	Our data were adapted from the study of Hill-Burns et al. All data are open access and de-identified. No ethical approval is required.

Note that full information on the approval of the study protocol must also be provided in the manuscript.

Field-specific reporting

Please select the one below that is the best fit for your research. If you are not sure, read the appropriate sections before making your selection.

☒ Life sciences ☐ Behavioural & social sciences ☐ Ecological, evolutionary & environmental sciences

For a reference copy of the document with all sections, see nature.com/documents/nr-reporting-summary-flat.pdf

Life sciences study design

All studies must disclose on these points even when the disclosure is negative.

Sample size	Data were adapted from the study of Hill-Burns et al
Data exclusions	no data were excluded from the analyses
Replication	confirm all attempts at replication were successful
Randomization	Data were adapted from the study of Hill-Burns et al
Blinding	Data were adapted from the study of Hill-Burns et al

Reporting for specific materials, systems and methods

We require information from authors about some types of materials, experimental systems and methods used in many studies. Here, indicate whether each material, system or method listed is relevant to your study. If you are not sure if a list item applies to your research, read the appropriate section before selecting a response.

Materials & experimental systems

n/a	Involved in the study
☒	☐ Antibodies
☒	☐ Eukaryotic cell lines
☒	☐ Palaeontology and archaeology
☒	☐ Animals and other organisms
☒	☐ Clinical data
☒	☐ Dual use research of concern
☒	☐ Plants

Methods

n/a	Involved in the study
☒	☐ ChIP-seq
☒	☐ Flow cytometry
☒	☐ MRI-based neuroimaging
